# Patient-Reported Outcome Measures (PROMs) Relevant to Musculoskeletal Conditions Translated and Validated in the Greek Language: A COSMIN-Based Systematic Review of Measurement Properties

**DOI:** 10.31138/mjr.32.3.200

**Published:** 2021-09-30

**Authors:** Ioannis Daskalakis, Ioannis Sperelakis, Barbara Sidiropoulou, Georgios Kontakis, Theodoros Tosounidis

**Affiliations:** 1 Department of Orthopaedic Surgery, University Hospital of Heraklion, Heraklion, Crete, Greece; 2 4th TO.M.Y., Heraklion, Crete, Greece

## Abstract

**Background:**

The use of patient-reported outcome measures (PROMs) constitutes a valuable tool in evaluating the quality of care offered in orthopaedic surgery. The aim of this review is to identify the PROMs that have been translated into and validated in the Greek language, summarise their measurement properties, and evaluate their methodological quality according to the COSMIN Risk of Bias Checklist.

**Methods:**

A structured literature search was conducted using the databases PubMED/MEDLINE, Embase, Scopus, and the Cochrane Library in order to identify PROMs relevant to musculoskeletal conditions translated and validated in the Greek language. The methodological quality of the studies was assessed according to the COSMIN Risk of Bias Checklist, and the quality of measurement properties according to the COMSIN criteria.

**Results:**

Literature search yielded 6743 articles. After removal of duplicates and screening of the articles, 32 studies including PROMs related to musculoskeletal conditions were identified. The studies included 31 PROMs and reported 171 measurement properties. Methodological quality was adequate for 81 of them (47.3%). The most commonly reported measurement properties were internal consistency, reliability, construct validity and responsiveness.

**Conclusion:**

The majority of PROMs translated into Greek involves the lower extremity and especially knee pathologies. The search revealed that there are areas of Musculoskeletal Medicine such as skeletal trauma, musculoskeletal oncology, and paediatric orthopaedics in which patient reported-outcome measures have not been translated into Greek. Translation and validation of new outcome measures is encouraged, using studies designed in compliance with the COSMIN guidelines, and further validation of the translated instruments.

## INTRODUCTION

Traumatic, degenerative, and inflammatory musculoskeletal conditions, are extremely common causes of pain and disability, that affect all patients’ age groups. They are responsible for a large number of health-care visits and days of hospitalisation, and many days of work loss.^1^ Proper clinical assessment and interpretation of imaging studies are crucial in order to achieve accurate diagnosis. However, during the treatment of musculoskeletal conditions, an important factor of decision-making is the impact of the disorder on the patient’s functional status and everyday activity. Therefore, it is necessary to evaluate the patient’s perspectives about their condition. The use of valid and reliable patient-reported outcome measures (PROMs) can offer better and more detailed assessment of the patient’s experience and provide critical information about prognosis and further management. Furthermore, more detailed and in-depth evaluation of patients’ experience is of paramount importance in order to achieve improvement of the provided care by the health-care facilities.

PROMs can be classified in three broad categories: generic, disease-specific, and condition-specific.^2^ Generic PROMs can be used for a broad spectrum of clinical conditions and measure single aspects of health or cover multiple dimensions of health status.^2^ Disease-specific PROMS are used to assess the outcome regarding a particular condition.^2^ Condition-specific PROMs are not used to assess a particular disease, but a broader health condition or state. They include a range of functional status or disability measures used to assess the health of a particular population group such as the elderly or those with mental health conditions.^3^ The selection of a PROM depends on the construct of interest and the measurement properties of the PROM.^4^ PROM measurement properties include reliability, validity and responsiveness.^5^ However, the quality of the studies providing evidence about the instruments’ measurement properties is often overlooked. The COSMIN (COnsensus-based Standards for the selection of health status Measurement INstruments) initiative developed a consensus-based standard for assessing the quality of studies on measurement properties.^5^

The purpose of PROM utilisation in clinical practice and research is to achieve an accurate representation of the patients’ perspectives. For that reason, it is important that the patient carries out the completion of the questionnaire unassisted. The inability of the patient to comprehend the questionnaire because of language difficulties can have a detrimental effect on the reliability of the data. As a result, translation of PROMs into other languages and cross-cultural adaptation using well-accepted methodological standards are necessary for the development of appropriate questionnaires. The aim of this review is to systematically identify the Greek-language validated PROMs reported in the published literature, which are used to assess musculoskeletal conditions and to evaluate the psychometric properties of the identified instruments using the COSMIN risk of bias checklist.

## METHODS

### Literature search

Structured search of Pubmed/MEDLINE, Embase, Scopus, and the Cochrane library was performed without time restriction, in order to identify studies translating and validating a PROM into the Greek language. Studies only in the English language were included. The electronic search was tailored to the individual database being searched and was based on the protocol suggested by the COSMIN group.^6^ The search strategy involved the combination of index terms and free-text words (including patient-reported outcome measures, quality of life, questionnaire, assessment tool, outcome tool, outcome measures, instrument, score, scale, cross cultural, Greek) and the Boolean operators ‘OR’ and ‘AND’. The final search was performed on 6 February 2021. Reference lists were hand-searched to identify potential additional relevant studies.

### Selection Criteria for Eligible Studies

After removal of duplicate studies, two reviewers (ID and IS) independently assessed all titles and abstracts. We included all studies that reported a translation and validation of at least one PROM, designed for the assessment of musculoskeletal conditions, into the Greek language. Clinical studies were eligible regardless of the presence or type of study intervention. Studies for de novo development of PROMs in Greek were also included. Any disagreement regarding eligibility of a study was resolved by consensus between the two reviewers, and if required, the senior author (T.T.) was consulted.

### Data Extraction

Data were extracted by ID and IS. The following data were extracted from each publication: the PROM, the intended construct for measurement, measurement properties, study population and diagnosis, number of patients, patient demographics, country and language.

### Assessment of the quality of studies and assessment of measurement properties

Two authors independently rated the methodological quality of the eligible studies using the COSMIN Risk of Bias checklist.^7^ Furthermore, the quality of measurement properties was assessed according to the COSMIN criteria for good measurement properties^6^

The COSMIN Risk of Bias checklist consists of 3 sections. The first section involves content validity, which is the degree to which the content of a PROM is an adequate reflection of the construct to be measured.^2^ Content validity evaluation includes: the relevance (all items in a PROM should be relevant for the construct of interest within a specific population and context of use), comprehensiveness (no key aspects of the construct should be missing), and comprehensibility (the items should be understood by patients as intended).^7^ In this systematic review, only the comprehensibility of the translated versions of PROMs was assessed, as relevance and comprehensiveness are considered more applicable to the initial development of the instrument. For the tools developed de novo in Greek, the development checklist was utilised and all components of content validity were evaluated. The second section of the checklist evaluates internal structure, and it consists of structural validity, internal consistency, and cross-cultural validity/measurement invariance. The third section involves the remaining measurement properties, which are: measurement error, criterion validity, hypotheses testing for construct validity and responsiveness. Each measurement property is awarded a score of “Very good”, “Adequate”, “Doubtful”, “Inadequate”, or not applicable. The methodological quality of each measurement property is assessed by a box containing questions scored on this scale according to defined COSMIN criteria. A system of ‘worst score counts’ applies for each box. The methodological quality of a measurement property could only be rated “Very good” if all the boxes of the checklist are rated “Very good”.

## RESULTS

### Search results

**Figure 1.**
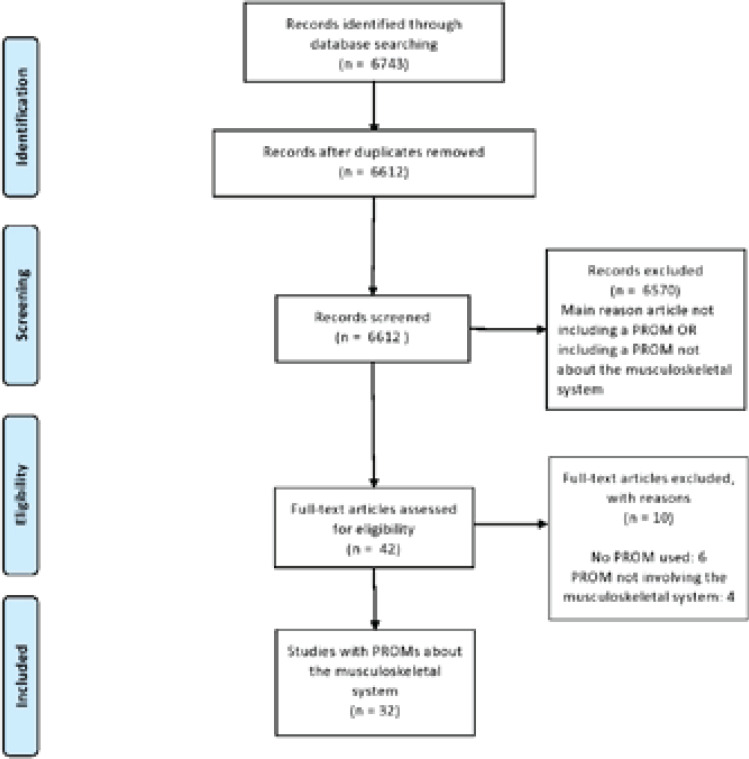


A total of 6743 studies were initially identified in the literature search. Removal of duplicates yielded 6612 studies. After screening, 43 full-text articles were retrieved, of which 32 met the inclusion criteria for this review. The study selection flow chart is shown in **Figure 1**.

The identified studies included 31 PROMs. Two of them were developed de novo in the Greek language, and 29 were translated versions. The characteristics of the identified studies are shown in **Table 1** and the characteristics of the identified PROMs are shown in **Table 2.**

**Table 1.** Characteristics of the translated PROMs validation studies.

**Study (year)**	**Instrument**	**Country (language)**	**Population (inclusion and exclusion criteria)**	**N**	**Mean age (SD, range)**	**Female: Male**
Stasi et al. (2020)^8^	mHHS	Greece (Greek)	Inclusion: existence of hip OA according to the Kellgren-Lawrence classification system. Exclusion: any kind of surgical intervention to the affected hip, other hip disorders or medical conditions, chronic inflammatory diseases or lower limb muscle weakness due to neurological aetiology, medication that adversely affected their postural or dynamic balance.	90	66.28 (8.27 55–87)	23:67
Stasi et al. (2020)^9^	iHOT-12	Greece (Greek)	Inclusion: existence of hip OA, according to the Kellgren–Lawrence classification system Exclusion: other types of arthritis, lower limb muscle weakness due to a central or peripheral neurological aetiology, insufficient knowledge of the Greek language.	124	65.80 (8.25, 50–85)	95:29
Papathanasiou et al. (2013)^10^	WOMAC	Greece (Greek)	Inclusion: existence of knee OA Exclusion: any kind of surgical intervention to the affected knee, medical conditions such as rheumatoid arthritis, psoriatic arthritis, systemic lupus erythematosus, lower limb muscle weakness due to a central or peripheral neurological aetiology, unstable angina, or uncontrolled hypertension or hypotension medication that adversely affected their postural or dynamic balance	123	69.5(6.2)	67:56
Konstantinidis et al. (2013)^11^	WOMAC Lequesne Index	Greece (Greek)	Inclusion: primary hip or knee OA, age greater than 40 years, ability to complete the questionnaires. Exclusion: secondary OA due to rheumatologic diseases, any other kinds of disabling lower limb arthro- or myopathy, physiotherapies of intra-articular perfusion or treatment with cortisone, diacerein or glucosamine in the last 6 months; symptoms of acute synovitis, of the affected joint, recent wound of injury of the lower leg, heavy respiratory or heart failure, peripheral vascular disease, severe psychiatric diseases, inability to speak Greek fluently.	97	Hip OA group: 67.66 ± 9.70 Knee OA group 69.46 ± 9.52	69:18
Kapreli et al. (2010)^12^	KOS-ADLS	Greece (Greek)	Inclusion: native Greek speakers Exclusion: pathological disorder or impairment involving both knees, other conditions that could affect lower extremity function	94	(24–61)	37:57
Moutzouri et al. (2014)^13^	KOOS	Greece (Greek)	Inclusion: over 40 years of age, fulfilment of criteria for primary TKR, ability to understand and complete the self-reported questionnaires. Exclusion: TKR for other reason than knee OA. significant disease in the lower limb or low back pain	60	72.1	44:16
Koumantakis et al. (2015)^14^	IKDC	Greece (Greek)	Inclusion: patients with knee disorders	80	35.3(11.9)	16:64
Moutzouri et al. (2020)^15^	KOOS-Child	Greece (Greek)	Inclusion: age 8–14 years, ability to understand and complete self-reported questionnaires, knee pathology symptoms confirmed by the orthopaedic surgeon’s clinical examination and medical history. Exclusion: cognitive impairments, congenital diseases, chronic illnesses, limited lower limb joints range of motion	59	11 (1.8)	30:29
Panagopoulos et al. (2020)^16^	LKSS TAS	Greece (Greek)	Inclusion: patients with various knee pathologies Exclusion: inflammatory or posttraumatic knee arthritis, infectious disease, age of <16 years, poor knowledge of Greek language, and the inability to understand and read Greek texts	55	24(7,17–54)	
Papadopoulos et al. (2016)^17^	AKPS	Greece (Greek)	Inclusion: age 18–45, anterior knee pain for at least 4 weeks Exclusion: age older than 45	130	20.1 (6.2, 18–45)	62:68
Korakakis et al. (2015)^18^	VISA-P	Greece (Greek)	Inclusion: age greater then 18, willing to participate in research, ability to give informed consent participation in sports. Exclusion: pregnancy and referred spinal symptoms.	187	26.3	
Touzopoulos et al. (2017)^19^	ATRS	Greece (Greek)	Inclusion: patients with acute Achilles’ tendon total rupture, treated surgically Exclusion: chronic ruptures, under 18 years old patients and traumatic bisections of Achilles tendon	46	41(10, 24–39)	4:42
Tsekoura et al. (2019)^20^	CAIT	Greece (Greek)	Inclusion: age greater than 18 years, ability to complete the self-reported questionnaires unassisted Exclusion: ankle sprain episode and/or lower extremity injury within the last 2 months	123	23.46(6.5, 20–29)	56:67
Kaoulla et al. (2008)^21^	MFPDI	Australia (Greek)	Inclusion: ability to walk household distances unaided	104	73.00, (5.26, 64–90)	64:40
Maliaropoulos et al. (2014)^22^	FASH	Greece (Greek, English, German)	Inclusion: activity in sport, age greater than 18 years Exclusion: pregnancy and referred spinal symptoms	140	22.8(3.6)	51:89
Korakakis et al. (2015)^23^	EILP	Greece (Greek)	Inclusion: age greater than 18 years, participation in sports Exclusion: pregnancy, previous lower extremity or spine surgery, referred pain from the lumbar region.	160	23.8 (4.4)	78:82
Vrouva et al. (2016)^24^	SPADI	Greece (Greek)	Inclusion: rotator cuff tear confirmed with US or MRI, conservative treatment Exclusion: neurological and Psychiatric conditions, surgery on the affected or the ipsilateral shoulder	102	47.4 (20–80)	60:42
Spanou et al. (2019)^25^	SPADI	Greece (Greek)	Inclusion: age greater than 18 years any kind of shoulder pain for at least four weeks Exclusion: cognitive, communication or psychological problems	130	44.5 (7.4)	68:62
Themistocleous et al. (2006)^26^	DASH	Greece (Greek)	Inclusion: Greek native language, unilateral disorder of the upper limb, healthy medical condition, informed consent, age above 18 years, ability to complete questionnaires independently, symptom duration of more than two months	106	46(20–68)	55:51
Bougea et al. (2018)^27^	BCTQ	Greece (Greek)	Inclusion: age equal to or greater than 18 years, first-time diagnosis of CTS, absence of severe intellectual disability or cognitive impairment Exclusion: symptoms, or signs of polyneuropathy, systemic diseases potentially associated with polyneuropathy, other diseases that cause hand symptoms, pregnancy	90	57.3(13.8, 23–88)	15:75
Goula et al. (2014)^28^	Hand20	Greece (Greek)	Inclusion: Greek native language, unilateral disorder of the upper limb, age above 18 years, ability to complete questionnaires independently, moderate to excellent general health condition	134	51.72(18–77)	92:42
Stasinopoulos et al. (2014)^29^	PRTEE	Greece (Greek)	Inclusion: clinical diagnosis of LET for at least 4 weeks Exclusion: dysfunction in the shoulder, neck and/or thoracic region, local or generalized arthritis, neurological deficit, radial nerve entrapment, limitations in arm functions, operative or conservative treatment for LET in the 4 weeks before entering the study	82	46.7 (18–60)	61:21
Graham et al. (2015)^30^	ASqOL	Greece (Greek)	Inclusion: confirmed AS diagnosis, aged 18 years and above, ability to understand and complete questionnaires independently, ability to provide written informed consent Exclusion: Presence of major co-morbidity with significant influence on subject’s QoL, inability to participate in the study due to cognitive disorders	92	49.6(11.5, 27–75)	29:63
Trouli et al. (2008)^31^	NDI	Greece (Greek)	Inclusion: age over 18, written consent of the patient, absence of symptoms below the elbows related to specific neck disorders. Exclusion: patients with symptoms below the elbow and one positive finding in the neurological testing and/or a positive Upper Limb Tension Test	65	62,3 (14,6)	29:36
Antonarakos et al. (2009)^32^	SRS-22	Greece (Greek)	Inclusion: patients with scoliosis, treated surgically	51	21,2 (16–27)	-
Potoupnis et al. (2012)^33^	SRS-22	Greece (Greek)	Inclusion: patients with idiopathic scoliosis, conservatively treated	87	(12–18)	80:7
Christakou et al. (2011)^34^	QBPDS	Greece (Greek)	Inclusion: low back pain lasting for at least 8 months age greater than 18 years old, adequate verbal ability and communication. Exclusion: significant anatomical abnormalities, the presence of inflammatory or neoplastic lesion, serious psychiatric disorders	130	41.5(11.6)	70:60
Boscainos et al. (2003)^35^	ODI RMDQ	Greece (Greek)	Inclusion: patients with low back pain	697	45.9(14.2,15–80)	235:462
Vasiladis et al. (2006)^36^	BrQ	Greece (Greek)	Inclusion: patients with idiopathic scoliosis, conservatively treated with brace	28	13.3	21:7
Zis et al. (2016)^37^	FiRST	Greece (Greek)	Inclusion: age greater than 18 years, duration of pain of more than 3 months, a native Greek speaker, willingness to provide written informed consent Exclusion: suffering from painful syndromes of other origins, gross cognitive deficits or intellectual disability, severe psychiatric comorbidity	101	Fibromyalgia group: 53.6 (11.9) Osteoarthritis group: 66.1 (14.1)	
Pratsidou et al. (2017)^38^	JAMAR	Greece (Greek)	Inclusion: patients with juvenile arthritis	375		269: 106

**Table 2.** Characteristics of the identified original PROMs that were translated and validated in Greek.

**PROM**	**Abbreviation**	**Year of Development**	**Original language**	**Intended construct and domains**	**Number of questions**	**Target of development population**
Modified Harris Hip Score	mHHS	2000^39^	English	Pain, function	8	Patients that underwent hip arthroscopy
International Hip Outcome Tool-12	iHOT-12	2012^42^	English	Symptoms and Functional Limitations, Sports and Recreational Activities Job-Related Concerns Social, Emotional, and Lifestyle Concerns	12	Patients with hip disorders
Western Ontario and McMaster Index	WOMAC	1988^43^	English	Pain, stiffness and physical functional disability	24	Patients with hip or knee osteoarthritis
Lequesne Algofunctional Index	-	1980^44^	English	Pain or discomfort, maximum walking distance with or without walking aids and physical/functional disability	11	Patients with hip osteoarthritis, on NSAIDs
Knee Outcome Survey- activities of Daily Living scale	KOS-ADLS	1998^45^	English	Symptoms/functional status in daily activities	17	Patients with knee disorders
Knee injury and Osteoarthritis Outcome score	KOOS	1998^46^	English	Pain, symptoms, activities of daily living, sport and recreation function, and knee-related quality of life	32	Patients that underwent ACL reconstruction
International Knee Documentation Committee Subjective Knee Form	IKDC	2001^47^	English	Symptoms, sports activities, function	10	Patients with knee disorders
Knee Injury and Osteoarthritis Outcome for Children	KOOS-Child	2012^48^	English, Swedish	Pain, symptoms, activities of daily living, sport and recreation function, and knee-related quality of life	32	Children with knee injuries
Lysholm Knee Scoring Scale	LKSS	1985^49^	English	Symptoms during daily activities	8	Patients with ACL injury
Tegner Activity Scale	TAS	1985^49^	English	Activity level in daily life and sports	10	Patients with ACL injury
Anterior knee pain scale (Kujala scale)	KAKPS	1993^50^	English	General symptoms/symptoms in sports activities	13	Patients with anterior knee pain and patellofemoral joint disorders
Victorian Institute of Sport Assessment scale- Patella	VISA-P	1998^51^	English	Symptoms, ability to participate in sports	10	Patients with patellar tendinopathy, asymptomatic controls
Achilles tendon rupture score	ATRS	2007^52^	English	Limitations of activity due to Achilles’ tendon rupture	10	Patients with Achilles tendon rupture
Cumberland Ankle Instability Tool	CAIT	2006^53^	English	Severity of functional ankle instability	9	Patients with ankle sprains
Manchester Foot Pain and Disability Index	MFPDI	2000^54^	English	Functional limitation, pain intensity, concern with personal appearance, difficulty in performing work or leisure activities	19	Patients with foot disorders, rheumatology patients
Functional Assessment Scale for Acute Hamstring Injuries	FASH	2014^22^	Greek, English, German	Pain during sports activities	10	Patients with hamstring injuries
Exercise Induced Leg Pain questionnaire	EILP	2012^55^	German	Symptoms/Difficulty in sports activities	10	Patients with exercise induced leg pain
Shoulder Pain and Disability Index	SPADI	1991^56^	English	Pain, disability	20	Patients with shoulder pain
Disabilities of the Arm, Shoulder and Head	DASH	1996^57^	English	Symptoms, physical function, social function	30	Patients with upper extremity conditions
Hand20	Hand20	2010^58^	English	Symptoms severity	20	Patients with upper extremity conditions
Boston carpal tunnel questionnaire	BCTQ	2018^59^	English	Symptoms severity, functional status	19	Patients with carpal tunnel syndrome
Patient-rated Tennis Elbow Evaluation	PRTEE	2005^60^	English	Pain, functional disability, difficulty in usual activities	15	Patients with lateral elbow tendinopathy
Ankylosing spondylitis Quality of Life	ASqOL	2003^61^	English, Dutch	Impact of ankylosing spondylitis on the quality of life	18	Patients with ankylosing spondylitis
Neck Disability Index	NDI	1991^62^	English	Symptoms in various activities	10	Patients with neck injuries
Scoliosis research society-22 questionnaire	SRS-22	2003^63^	English	Function/activity, Physical functioning Pain, Role physical, Mental health, Bodily pain, Self-image/appearance, General health, Satisfaction with management	22	Patients with scoliosis
Quebec Back Pain Disability Scale	QBPDS	1996^64^	English, French	Functional disability during: rest/bed, sit/stand, ambulation, handling of large/heavy objects, movement and bending/stooping	20	Patients with back pain
Oswestry Disability Index	ODI	1980^65^	English	Pain Intensity, personal care, lifting	10	Patients with back pain
Roland-Morris Disability Questionnaire	RMDQ	1983^66^	English	Function	24	Patients with back pain
BRACE questionnaire	BrQ	2006^36^	Greek	General health perception, physical functioning, emotional functioning, self-esteem and aesthetics, vitality, school activity, bodily pain and social functioning	34	Patients with idiopathic scoliosis treated conservatively
Fibromyalgia Rapid Screening Tool	FiRST	2010^67^	English, French	Pain	6	Patients with chronic pain due to fibromyalgia
Juvenile Arthritis Multidimensional Assessment Report	JAMAR	2011^68^	English	Overall well-being, functional status, health-related quality of life (HRQoL), pain, morning stiffness, disease activity/status/course, articular and extra-articular involvement, drug-related side effects/compliance, and satisfaction with illness outcome.	38	Patients with juvenile arthritis

All the instruments that were identified regarding the musculoskeletal system were disease-specific. The majority of the questionnaires (16) involved the lower limb.^8–23^ Nine of them involved knee conditions,^10–18^ which was the entity with the largest number of PROMs translated into the Greek language. Four instruments were retrieved for the evaluation of hip conditions,^8–10^ while three questionnaires involved foot and ankle pathologies.^19–21^ Five instruments were retrieved about upper limb conditions tools translated and validated in Greek.^24–29^ Regarding spine conditions, six instruments were retrieved,^30–35^ with two of them being health-related quality of life measures.^30,32,33^ Two questionnaires involved other conditions: fibromyalgia^37^ and juvenile arthritis.^38^ Two questionnaires that were constructed de novo in Greek were also retrieved, the Functional Assessment Scale for Acute Hamstring Injuries score (FASH score)^22^ and the Brace questionnaire (BrQ).^36^

### Quality of the included studies

In 32 identified studies, 31 PROMs were validated. The total number of reported measurement properties was 171. The methodological quality for 37 of them (21%) was inadequate and doubtful for 43 (25%) of them. Many measurement properties were not reported. The methodological quality of the studies is summarized at **Tables 3 and 4**. The measurement properties of each PROM were rated according to the COSMIN criteria for good measurement properties (**Table 5**).

**Table 3.** Comprehensibility assessment of the translated versions of PROMs.

**PROM**	**Methodological quality**	**Rating**
mHHS^8^	Doubtful	-
iHOT-12 ^9^	Doubtful	-
WOMAC ^10^		
Lequesne Index^10^	Doubtful	-
WOMAC^11^	Doubtful	-
KOS-ADLS^12^	Doubtful	-
KOOS^13^	Doubtful	-
IKDC^14^	Doubtful	-
KOOS-Child^15^	Doubtful	-
LKSS^16^	Adequate	+
TAS^16^	Adequate	+
AKPS^17^	Doubtful	-
VISA-P^18^	Doubtful	-
ATRS^19^		
CAIT^20^		
MFPDI^21^		
FASH^22^	Inadequate	-
EILP^23^	Doubtful	-
SPADI^24^	Doubtful	-
SPADI^25^	Doubtful	-
DASH^26^		
BCTQ^27^		
Hand20^28^		
PRTEE^29^	Doubtful	
ASqOL^30^	Adequate	+
NDI^31^	Doubtful	
SRS-22^32^	Doubtful	
SRS-22^33^	Doubtful	
QBPDS^34^	Adequate	+
ODI^35^	Doubtful	
RMDQ^36^	Doubtful	
BrQ^37^		
FiRST^38^	Doubtful	
JAMAR^39^	Doubtful	

**Table 4.** Methodological quality of the translated versions’ validation studies per measurement property.

**Instrument**	**Structural validity**	**Internal consistency**	**Cross-cultural validity**	**Reliability**	**Measurement error**	**Criterion validity**	**Hypotheses testing for construct validity**	**Responsiveness**
mHHS^8^		Very good		Inadequate	Inadequate		Adequate	Doubtful
iHOT-12 ^9^	Very good	Very good		Adequate	Adequate	Very good	Very good	Very good
WOMAC ^10^	Adequate	Very good		Inadequate			Adequate	Adequate
Lequesne Index^10^	Inadequate	Very good		Inadequate	Inadequate		Doubtful	Doubtful
WOMAC^11^	Inadequate	Very good		Inadequate	Inadequate		Doubtful	Doubtful
KOS-ADLS^12^	Adequate	Adequate		Inadequate	Inadequate		Adequate	Very good
KOOS^13^		Very good		Adequate	Adequate		Adequate	Adequate
IKDC^14^		Doubtful		Adequate	Adequate		Adequate	Adequate
KOOS-Child^15^	Inadequate	Very good		Adequate	Adequate		Adequate	Adequate
LKSS^16^		Very good		Inadequate	Inadequate		Adequate	Adequate
TAS^16^		Very good		Inadequate	Inadequate		Adequate	Adequate
AKPS^17^		Very good		Adequate	Adequate		Adequate	Adequate
VISA-P^18^	Adequate	Very good		Adequate	Adequate		Adequate	Inadequate
ATRS^19^		Very good		Adequate			Adequate	Adequate
CAIT^20^	Inadequate	Very good	Adequate	Inadequate			Very good	Adequate
MFPDI^21^		Very good					Adequate	Adequate
FASH^22^	Adequate	Very good		Inadequate	Inadequate		Adequate	Adequate
EILP^23^	Adequate	Inadequate		Inadequate	Inadequate		Very good	Very good
SPADI^24^	Adequate	Very good	Inadequate	Inadequate			Adequate	Adequate
SPADI^25^		Very good		Inadequate	Inadequate		Adequate	Adequate
DASH^26^	Inadequate	Doubtful		Inadequate			Adequate	Adequate
BCTQ^27^		Very good		Inadequate	Inadequate	Very good	Adequate	Adequate
Hand20^28^		Very good		Inadequate			Adequate	Adequate
PRTEE^29^		Doubtful		Doubtful	Doubtful		Adequate	Adequate
ASqOL^30^	Doubtful	Very good		Doubtful	Doubtful		Adequate	Adequate
NDI^31^	Adequate	Doubtful		Doubtful	Doubtful		Very good	Very good
SRS-22^32^	Inadequate	Very good		Doubtful			Adequate	Adequate
SRS-22^33^		Very good		Doubtful			Adequate	Adequate
QBPDS^34^		Very good					Adequate	Adequate
ODI^35^		Very good					Adequate	Adequate
RMDQ^36^	Adequate	Very good					Adequate	Adequate
BrQ^37^		Very good					Adequate	Adequate
FiRST^38^	Adequate	Very good		Inadequate		Very good	Adequate	Very good
JAMAR^39^	Adequate	Very good		Doubtful		Very good	Adequate	Adequate

**Table 5.** Quality of the translated versions’ measurement properties.

**Instrument**	**Structural validity**	**Internal consistency**	**Cross-cultural validity**	**Reliability**	**Measurement error**	**Criterion validity**	**Hypotheses testing for construct validity**	**Responsiveness**
mHHS^8^	N/R	-	N/R	+	?	N/R	?	?
iHOT-12 ^9^	?	+	N/R	+	?	?	?	?
WOMAC ^10^	N/R	+	N/R	+	N/R	?	?	?
Lequesne Index^10^	N/R	+	N/R	+	?	?	?	?
WOMAC^11^	N/R	+	N/R	+	?	?	?	?
KOS-ADLS^12^	N/R	+	N/R	+	?	N/R	+	+
KOOS^13^	N/R	+	N/R	+	?	N/R	+	+
IKDC^14^	N/R	+	N/R	+	?	N/R	+	+
KOOS-Child^15^	?	+	N/R	+	?	N/R	+	+
LKSS^16^	N/R	+	N/R	+	?	N/R	?	?
TAS^16^	N/R	+	N/R	+	?	N/R	?	?
AKPS^17^	N/R	+	N/R	+	?	N/R	?	?
VISA-P^18^	?	+	N/R	+	?	N/R	+	+
ATRS^19^	N/R	+	N/R	+	N/R	N/R	+	+
CAIT^20^	N/R	+	?	+	N/R	N/R	?	?
MFPDI^21^	N/R	+	N/R	N/R	N/R	N/R	?	?
FASH^22^	?	?	N/R	+	+	N/R	?	?
EILP^23^	?	?	N/R	+	+	N/R	?	?
SPADI^24^	+	+	+	+	N/R	N/R	+	+
SPADI^25^	N/R	+	N/R	+	?	N/R	+	+
DASH^26^	?	+	N/R	-	N/R	N/R	?	?
BCTQ^27^	N/R	+	N/R	+	N/R	+	+	+
Hand20^28^	N/R	+	N/R	+	N/R	N/R	?	?
PRTEE^29^	N/R	?	N/R	+	-	N/R	?	?
ASqOL^30^	N/R	+	N/R	?	N/R	N/R	?	?
NDI^31^	-	?	N/R	+	+	N/R	?	?
SRS-22^32^	N/R	+	N/R	+	N/R	NA	?	?
SRS-22^33^	?	+	N/R	+	N/R	N/R	?	?
QBPDS^34^	N/R	+	N/R	N/R	N/R	N/R	?	?
ODI^35^	N/R	+	N/R	N/R	N/R	N/R	?	?
RMDQ^36^	?	+	N/R	N/R	N/R	N/R	?	?
BrQ^37^	N/R	?	N/R	N/R	N/R	N/R	?	?
FiRST^38^	N/R	+	N/R	+	N/R	+	+	+
JAMAR^39^	N/R	+	N/R	-	N/R	N/R	?	?

### Summary of translated PROMs

#### PROMs about hip disorders

The modified Harris Hip Score was developed in 2000,^39^ as a modification of the original Harris Hip Score.^40^ It includes only assessments about pain and function, therefore it can be used as a patient-reported outcome measure. Reliability of The Greek version of mHHS^8^ received sufficient rating. The rest of the measurement properties were indeterminate or were not reported.

The 12-item International Hip Outcome Tool (iHOT-12)^41^ was developed as a shorter version of the 33-item International Hip Outcome Tool questionnaire,^42^ and it is used for the assessment of the quality of life of patients of hip disorders. In the Greek version of iHOT-12, reliability was rated sufficient. The rest of the measurement properties were indeterminate or were not reported.

#### PROMs about knee disorders

Literature search yielded nine instruments for the evaluation of knee conditions translated in Greek. The Western Ontario and McMaster Osteoarthritis Index (WOMAC) is a 24-item questionnaire,^43^ designed for the assessment of patients with hip or knee osteoarthritis. It has been translated and validated in Greek in two studies.^10,11^ In the study of Konstantinidis et al.,^11^ comparative validation with the Lequesne Index^44^ was performed. The majority of the participants (68 of 97) were patients with knee osteoarthritis, with the rest being patients with hip osteoarthritis. The study of Papathanasiou et al.^11^ included only patients with knee osteoarthritis. In both studies, internal consistency and reliability received sufficient ratings. The rest of the measurement properties received adequate ratings.

Six instruments were retrieved that can be used for various knee pathologies, the Knee Outcome Survey-Activities of Daily Living Scale (KOS-ADLS), ^12^ the Knee Injury and Osteoarthritis Outcome Score (KOOS)^13^ and KOOS-Child,^14^ the International Knee Documentation Committee Subjective Knee Form (IKDC)^15^, the Lysholm Knee Scoring Scale (LKSS),^16^ and the Tegner Activity Scale (TAS).^16^

The KOS-ADLS is a 14-item questionnaire assessing the symptoms and function during daily activities of patients with knee pathologies.^45^ The Greek version of KOSADLS^12^ received sufficient ratings for internal consistency, reliability, construct validity and responsiveness. The rest of the measurement properties were not reported or indeterminate.

The KOOS consists of 42 questions divided into five domains.^46^ It assesses Pain (9 items), Symptoms (7 items), Activity of Daily Living (ADL; 17 items), Sport and Recreation Function (Sports/Rec; 5 items) and Quality of Life (QoL; 4 items). The Greek version of KOOS^13^ received sufficient ratings for internal consistency, reliability, construct validity and responsiveness. The rest of the measurement properties were not reported or were indeterminate. Due to difficulty of understanding some of the items by the paediatric population, another version of KOOS was developed, modified for children.^47^ Reliability, construct validity and responsiveness of the Greek version were rated sufficient. All other measurement properties were indeterminate or were not reported.

The IKDC^48^ is a 10-item instrument and evaluates symptoms and functional status of both daily life and sports activities, The Greek version of IKDC^14^ received sufficient ratings for internal consistency, reliability, construct validity, and responsiveness. The rest of the measurement properties were not reported or indeterminate.

The internal consistency and reliability of the Greek versions were rated sufficient. All other measurement properties were indeterminate or not reported.

Two instruments were retrieved that were specifically designed for the assessment of anterior knee pain, the Kujala Anterior Knee Pain Scale (KAKPS),^17^ and the Victorian Institute of Sport Assessment scale-Patella (VISA-P) questionnaire.^18^ All measurement properties of the KAKPS Greek version^17^ were indeterminate or not reported, except for internal consistency and reliability. Regarding the measurement properties of the VISA-P Greek version,^18^ they were indeterminate or not reported, besides construct validity and responsiveness.

#### PROMs about ankle disorders

The Achilles Tendon Rupture Score (ATRS) is the only outcome measure validated for Achilles’ tendon ruptures.^52^ Its purpose is the evaluation of symptoms and function after Achilles tendon rupture. Internal consistency, reliability, construct validity and responsiveness of the Greek version^19^ received sufficient ratings. The rest were not reported.

The Cumberland Ankle Instability Tool (CAIT) is a questionnaire of nine independently-scored items, for the assessment of symptoms of ankle instability.^53^ The Greek version of the CAIT^20^ received sufficient ratings for internal consistency and reliability. All other measurement properties were indeterminate or were not reported.

The (Manchester Foot and Pain Disability Index) MFPDI was the only retrieved tool translated and validated in Greek^21^ that is designed for the assessment of disability caused by foot disorders. It consists of 19 items, that starting with the statement “Because of pain in my feet”, divided in three subscales: functional limitation, pain intensity, concern with personal appearance. Only internal consistency of the Greek version was rated sufficient. Reliability and responsiveness were indeterminate. All other measurement properties were not reported.

#### PROMs about upper limb disorders

The Shoulder Pain and Disability Index has been translated and validated in Greek in two studies.^24,25^ In the study of Vrouva et al.,^24^ the participants were patients with rotator cuff tear, treated conservatively. All measurement properties were rated sufficient except for measurement error and criterion validity that were not reported. In the study of Spanou et al.,^25^ the participants were patients that suffered of shoulder pain for at least four weeks. Internal consistency, construct validity and responsiveness were rated sufficient. All other measurement properties were indeterminate or not reported.

The Disabilities of the Arm, Shoulder, and Hand (DASH) Questionnaire is utilized for the assessment of a variety of symptoms associated with upper limb disorders. Only internal consistency of the Greek version^26^ received sufficient rating. Reliability was rated insufficiently, and the rest of the measurement properties were indeterminate or not reported. The Hand20 questionnaire was also designed for the assessment of a variety of symptoms of upper limb disorders.^58^ Evaluation of the measurement properties’ quality showed sufficient internal consistency and reliability, with all other measurement properties being indeterminate or not reported.

One instrument was retrieved for the evaluation of hand conditions, the Boston Carpal Tunnel Questionnaire (BCTQ).^59^ All measurement properties of the Greek version^28^ received sufficient ratings, except for structural validity, cross-cultural validity and measurement error that were not reported.

One questionnaire was retrieved about elbow disorders, the Patient-rated Tennis Elbow Evaluation (PRTEE) which is an updated version of the Patient-Rated Forearm Evaluation Questionnaire (PRFEQ).^60^ The Greek version of PRTEE^29^ received sufficient rating for reliability. All other measurement properties were indeterminate or not reported.

#### PROMs about spine disorders

Six instruments were retrieved for the evaluation of spine conditions. The Neck Disability Index is a short, condition-specific questionnaire used for patients with neck pain.^61^ It consists of 10 items concerning various activities. The Greek version of NDI^31^ received sufficient ratings for reliability and measurement error. All other measurement properties were indeterminate or not reported.

Regarding the assessment of patients with low back pain, three condition-specific tools were identified: the Quebec Back Pain Disability Scale (QBPDS),^34^ the Oswestry Disability Index (ODI) and the Roland-Morris Disability Questionnaire (RMDQ).^35^ Internal consistency of all three translated versions was rated sufficient. All other measurement properties were indeterminate or not reported.

Finally, two patient reported Health-Related quality of Life measures (HRQoL) were identified: the Ankylosing Spondylitis Quality of Life (ASQoL) questionnaire^30^ and the scoliosis research society – 22 (SRS-22) questionnaire.^32,33^ The internal consistency of the ASQoL Greek version^30^ was rated sufficient. All other measurement properties were indeterminate or not reported. The SRS-22 has been translated and validated in Greek in two studies. The study of Antonarakos et al.^32^ included surgically treated patients, while the study of Potoupnis et al.^33^ included conservatively treated patients. The ratings were similar: sufficient internal consistency and reliability, with the rest of the measurement properties being indeterminate or not reported.

#### PROMs constructed de novo in Greek

Two instruments were retrieved that were constructed de novo in Greek, the Brace questionnaire (BrQ) and the Functional Assessment Scale for Acute Hamstring Injuries (FASH). The BrQ was constructed by Vasiliadis et al. in 2006^36^ and it is a HRQoL measure for adolescents with idiopathic scoliosis treated conservatively. It consists of 34 items divided in 8 subdomains. The methodology for total PROM design received “inadequate” rating, due to the fact that the construct of interest was not clearly described according to the COSMIN criteria.^7^ Pilot test of the questionnaire was not performed, therefore the content validity of the questionnaire was not assessed. The FASH questionnaire was constructed in 2014 by Malliaropoulos et al.^22^ It is a condition-specific, 10-item questionnaire designed to evaluate the functional status of athletes with hamstring injuries. Total PROM design was rated “inadequate”, as the description of the construct was not clear. Pilot test was performed, and the sample was an accurate representation of the target population. However, the items were not tested in their final form; thus, the methodological quality of comprehensibility assessment was rated “inadequate”. Comprehensiveness was not assessed. Summary of PROM development checklist is presented in **Table 6**.

**Table 6.** Quality of development of PROMs that were constructed de novo in Greek.

**PROM**	**PROM design**							**Pilot test**			**Total PROM development**
	Clear construct	Clear origin of construct	Clear target population for which the PROM was developed	Clear context of use	PROM developed in sample representing the target population	Concept elicitation	Total PROM design	Pilot test performed in sample representing the target population	Comprehensibility	Comprehensiveness	
FASH^22^	Inadequate	Doubtful	Very good	Very good	Very good	Adequate	Inadequate	Very good	Inadequate		Inadequate
BrQ^36^	Inadequate	Doubtful	Very good	Very good	Very good	Doubtful	Inadequate	NA	NA	NA	Inadequate

## DISCUSSION

The purpose of this review was to summarise the PROMs involving musculoskeletal conditions that have been translated and validated in Greek, and to also evaluate the methodological quality of the validation studies according to the COSMIN Risk of Bias Checklist.^7^ Thirty-one translated versions of PROMs were identified. The methodological quality for 47,3% (n=81) of the measurement properties was adequate and 45% (n=77) of the measurement properties received the “sufficient” rating. The remaining measurement properties were indeterminate or not reported.

Content validity is the degree to which the content of an instrument is an adequate reflection of the construct to be measured^5^ and is the most important measurement property of a PROM. Comprehensibility is a significant component of content validity, and it was rated “insufficient” in the majority of the studies, as cognitive debriefing was not performed during pre-testing or the process that was used was not clearly described. Other components of content validity (comprehensiveness and relevance) were not evaluated in this systematic review, as they are considered more applicable to the initial development of a PROM.

Structural validity refers to the degree to which the scores of a PROM are an adequate reflection of the dimensionality of the construct to be measured.^3^ It is usually assessed with factor analysis. In the majority of the studies, factor analysis was not performed,^8,11,14,15,16–19,20,24,26–28,31–33,36^ and the authors assumed models from other studies that evaluated the structural validity of the construct of interest.

Internal consistency is an important component of internal structure of a PROM. It represents the degree of interrelatedness among the items and is often assessed by Cronbach’s alpha.^5^ For the appropriate interpretation of internal consistency, the items should form a unidimensional scale or subscale. Unidimensionality means that the items in a scale or a subscale measure a single construct. Internal consistency was one of the most frequently reported measurement properties across the studies. The methodological quality was sound in the vast majority of them, with the calculation of Cronbach’s alpha.

Cross cultural adaptation is the cornerstone of the comprehensibility of a PROM, and it is absolutely necessary for the accurate reflection of a PROM in another language. The translation process in most of the studies was in compliance with the international guidelines (such as those of Beaton et al.^69^). For further confirmation of cross-cultural validity, it is suggested by COSMIN guidelines to perform comparisons between at least two different groups, with differences such as gender, literacy or language. However, such comparisons were performed only in two studies, the validation of CAIT^20^ and the validation of SPADI by Vrouva et al.^24^

Reliability refers to the total variance in the measurements which is due to “true” differences between patients. “True” is the average score that would be obtained if the scale was administered an infinite number of times to the same person.^5^ It does not concern the accuracy of an instrument, but only its consistency.^70^ Reliability also refers to the ability of a PROM to distinguish between patients.^5^ Reliability was reported in 27 of 32 studies. The methodology was inadequate in 20 them, even though the results were sufficient (ICC >70). The main reason for the inadequate rating of methodology was that the interval between the first and the second completion of the questionnaire was much shorter than 2 weeks that is deemed acceptable by the COSMIN guidelines.^7^ The same shortcoming applied and for measurement error calculation.

Hypotheses testing for construct validity refers to the consistency of the scores of a PROM with a hypotheses, assuming that the PROM validly measures the construct to be measured. The more specific the hypotheses are and the more hypotheses are being tested, the more evidence is gathered for construct validity.^71^ Many types of hypotheses can be tested to evaluate construct validity of a PROM.^5^ Responsiveness refers to the ability of a PROM to detect change over time in the construct to be measured. The difference between construct validity and responsiveness is that construct validity refers to the validity of a single score, and responsiveness refers to the validity of a change score.^5^ The standards for evaluation of responsiveness are similar to the standards utilised for construct validity evaluation. Hypotheses testing for construct validity and responsiveness were reported in all the studies. The most common method of assessment was comparison with other outcome measures and the methodological quality was adequate. However, in most of the studies, the results were deemed indeterminate due to the lack of an a priori hypotheses statement.

Literature search did not yield any further validation studies for any of the translated versions of PROMs, besides the initial ones. However, it cannot be excluded that the instruments are utilized in everyday clinical practice. The BrQ^36^ has been translated and validated in Polish,^71^ Italian,^72^ French,^73^ Korean,^74^ and Persian.^75^ The results of the validation studies were satisfactory regarding reliability. The FASH scale has been validated in German^76^ and French.^77^ The validation studies reported satisfactory internal consistency and reliability results.

To the best of our knowledge, this is the first systematic review that summarises the PROMs related to the musculoskeletal system that have been translated in the Greek language and evaluates their measurement properties according to the COSMIN criteria. This review can be used as an everyday clinical practice reference guide for clinicians, in relation to the available instruments translated in the Greek language. It also highlights the strengths and limitations of the studies conducted with the aim of PROM validation in the Greek language. Therefore, it offers information for future researchers in relation to the quality of the existing studies and how to avoid shortcomings in the future. The limitation of this study is that it only includes studies with PROMs constructed in the English language. Instruments constructed in other languages were not included.

Literature search for this review revealed that there is a lack of translated and validated instruments in Greek in several areas of musculoskeletal medicine, such as traumatology, paediatric orthopaedics, and orthopaedic oncology. Further research is encouraged with studies in compliance with the COSMIN criteria in order to translate and validate new outcome measures in Greek regarding those areas. In addition, further research is encouraged regarding the PROMs that have already been translated in Greek, in order to achieve further validation of their measurement properties and report the measurement properties that have not been previously reported.

## CONCLUSION

A number of PROMs has been translated into the Greek language related to musculoskeletal conditions. The majority of them involves the lower limb and especially knee conditions. Further validation of these instruments is encouraged, with studies of good quality according to the COSMIN checklist. In addition, there are quite a few fields of musculoskeletal medicine where outcome measures have not been translated yet. Therefore, it is indicated that new tools need to be translated into Greek, in compliance with the COSMIN criteria that will involve those areas of clinical practice.
